# The effect of home-based transcranial direct current stimulation in cognitive performance in fibromyalgia: A randomized, double-blind sham-controlled trial

**DOI:** 10.3389/fnhum.2022.992742

**Published:** 2022-11-24

**Authors:** Paul Vicuña Serrano, Maxciel Zortea, Rael Lopes Alves, Gerardo Beltrán, Cibely Bavaresco, Leticia Ramalho, Camila Fernanda da Silveira Alves, Liciane Medeiros, Paulo R. S. Sanches, Danton P. Silva, Iraci Lucena da Silva Torres, Felipe Fregni, Wolnei Caumo

**Affiliations:** ^1^Post-Graduate Program in Medical Sciences, School of Medicine, Universidade Federal do Rio Grande do Sul (UFRGS), Porto Alegre, Brazil; ^2^Laboratory of Pain and Neuromodulation at Hospital de Clínicas de Porto Alegre (HCPA), Porto Alegre, Brazil; ^3^Centro Universitário Cesuca, Cachoeirinha, Brazil; ^4^Institute of Neurosciences of the Universidad Catolica de Cuenca, UCACUE, Cuenca, Ecuador; ^5^Programa de Pós-Graduação em Saúde e Desenvolvimento Humano, Universidade La Salle, Canoas, Brazil; ^6^Laboratory of Biomedical Engineer at HCPA, Porto Alegre, Brazil; ^7^Pain and Palliative Care Service at HCPA, Porto Alegre, Brazil; ^8^Laboratorio de Farmacologia da Dor e Neuromodulação: Investigacoes Pre-clinicas, Centro de Pesquisa Experimental (CPE), Hospital de Clínicas de Porto Alegre (HCPA), Porto Alegre, Brazil; ^9^Laboratory of Neuromodulation, Department of Physics and Rehabilitation, Center for Clinical Research Learning, Spaulding Rehabilitation Hospital, Boston, MA, United States; ^10^Department of Surgery, School of Medicine, Universidade Federal do Rio Grande do Sul (UFRGS), Porto Alegre, Brazil; ^11^School of Medicine, Universidade Federal do Rio Grande do Sul (UFRGS), Porto Alegre, Brazil

**Keywords:** fibromyalgia, pain, cognition, working memory, tDCS

## Abstract

**Background:**

Transcranial Direct Current Stimulation (tDCS) is a promising approach to improving fibromyalgia (FM) symptoms, including cognitive impairment. So, we evaluated the efficacy and safety of home-based tDCS in treating cognitive impairment. Besides, we explored if the severity of dysfunction of the Descendant Pain Modulation System (DPMS) predicts the tDCS effect and if its effect is linked to changes in neuroplasticity as measured by the brain-derived neurotrophic factor (BDNF).

**Methods:**

This randomized, double-blind, parallel, sham-controlled clinical trial, single-center, included 36 women with FM, aged from 30 to 65 years old, assigned 2:1 to receive a-tDCS (*n* = 24) and s-tDCS (*n* = 12). The primary outcome was the Trail Making Test’s assessment of executive attention, divided attention, working memory (WM), and cognitive flexibility (TMT-B-A). The secondary outcomes were the Controlled Oral Word Association Test (COWAT), the WM by Digits subtest from the Wechsler Adult Intelligence Scale (WAIS-III), and quality of life. Twenty-minute daily sessions of home-based tDCS for 4 weeks (total of 20 sessions), 2 mA anodal-left (F3) and cathodal-right (F4) prefrontal stimulation with 35 cm^2^ carbon electrodes.

**Results:**

GLM showed a main effect for treatment in the TMT-B-A [Wald χ2 = 6.176; Df = 1; *P* = 0.03]. The a-tDCS improved cognitive performance. The effect size estimated by Cohen’s d at treatment end in the TMT-B-A scores was large [–1.48, confidence interval (CI) 95% = –2.07 to–0.90]. Likewise, the a-tDCS effects compared to s-tDCS improved performance in the WM, verbal and phonemic fluency, and quality-of-life scale. The impact of a-tDCS on the cognitive tests was positively correlated with the reduction in serum BDNF from baseline to treatment end. Besides, the decrease in the serum BDNF was positively associated with improving the quality of life due to FM symptoms.

**Conclusion:**

These findings revealed that daily treatment with a home-based tDCS device over l-DLPFC compared to sham stimulation over 4 weeks improved the cognitive impairment in FM. The a-tDCS at home was well-tolerated, underlining its potential as an alternative treatment for cognitive dysfunction. Besides, the a-tDCS effect is related to the severity of DPMS dysfunction and changes in neuroplasticity state.

**Clinical trial registration:**

[www.ClinicalTrials.gov], identifier [NCT03843203].

## Introduction

Fibromyalgia (FM) comprises widespread chronic pain and concurs with significant emotional distress associated with functional disability for daily activities. The symptoms linger or recur for at least 3 months without other conditions explaining the pain (Treede et al., 2015; Dueñas et al., 2016). The symptoms’ severity scale of the American College of Rheumatology (ACR, 2016) diagnosis criteria included cognitive impairment as an element of core symptoms of FM (Montoro et al., 2015). Attention, perception, memory, executive functioning, and language abilities are essential components of cognition (Gellman and Rick Turner, 2013). The processing of cognitive components includes active decision-making, learning, and memory of past events (Hansen and Streltzer, 2005; Moriarty et al., 2011). This complex processing involves extensive cortical and subcortical neural circuitry responsible for perception, localization, processing, relaying, and pain modulation. Thus, the pain experience is modulated by affective-motivational and cognitive-evaluative components than being a purely sensory phenomenon (Tyng et al., 2017). Chronic pain syndromes, such as FM, have been linked to cognitive processing disturbance (Khera and Rangasamy, 2021).

There is evidence that pain and neurocognition have anatomical, biochemical, and molecular associations (Khera and Rangasamy, 2021). The frontal lobes control executive functions, particularly the orbitofrontal cortex, anterior cingulate cortex, and dorsolateral prefrontal cortex (DLPC) (Verdejo-García et al., 2009). The somatosensory cortex distinguishes between painful and non-painful sensations, whereas the medial thalamus and anterior cingulate cortex (ACC) record the stimuli as painful. The emotional component of pain perception and memory formation are both impacted by this encoding process, which is also linked to improved functional connectivity between the thalamus and the mPFC (Tseng et al., 2017). There is an overlap of brain structures involved in executive function and pain perception, and either cognitive impairment or chronic pain involves maladaptive neuroplasticity processes (Khera and Rangasamy, 2021). Higher executive functioning requires the ability to make emotional decisions (Tyng et al., 2017). They include executive function, learning, memory, sustained focus, processing speed, and psychomotor ability (Khera and Rangasamy, 2021). The cognitive impairment hinders interaction with the environment and generates difficulties with working memory (WM) (Miller et al., 2018). The WM is responsible for the temporary storage and manipulation of information necessary to perform complex tasks, such as language comprehension, learning, and reasoning (Cowan, 2014). It is essential to the adequate performance of complex behaviors. Hence, when it fails, so does the capacity to carry out daily living activities and the ability to elaborate pain confrontation strategies (D’Esposito and Postle, 2015). In FM, the core complaints related to cognitive impairment are mental confusion, concentration difficulties, and failing memory. This set of symptoms is often called “FibroFog” (Kravitz and Katz, 2015; Walitt et al., 2016; Bell et al., 2018). According to a recent study, FM patients performed less accurately on activities requiring split attention and attentional switching (Moore et al., 2019). Regardless of chronic pain impact on cognitive impairment in FM, it does not seem to correlate with other musculoskeletal or neuropathic pain (Grisart and Van der Linden, 2001; Verdejo-García et al., 2009).

Clinical and preclinical studies indicate a bidirectional link between cognition and chronic pain (Serrano et al., 2022). However, the targets of treatment of chronic pain comprise modulation of the central sensory processing either pain transmission [i.e., opioids and tricyclic antidepressants (TCAs) or neural excitability should be the therapeutic targets (i.e., opioids, anticonvulsants)]. However, multiple medicines are needed to treat FM symptoms; some might worsen cognitive impairment (i.e., opioids) (Ngian et al., 2011). Despite the modulation of the central sensory pain processing to be a treatment target, the pharmacological approaches might be ineffective in many patients (Schiltenwolf et al., 2014), and some of them can worsen cognitive performance, so the interest in non-pharmacological interventions. Among these interventions, transcranial direct current stimulation (tDCS) has demonstrated clinical benefits for complex chronic pain conditions, such as FM (Zortea et al., 2019). The main target to apply the anodal(a)-tDCS for pain is the primary motor cortex (M1), based on the rationale that it enhances the excitability of the sensory-discriminative networks (Zortea et al., 2019). Another potential target area to apply the a-tDCS is over DLPFC since it has been found to have beneficial effects on mood regulation, cognitive functions, and maladaptive emotional functioning (Dixon et al., 2017; Sankarasubramanian et al., 2017). Regarding the a-tDCS impact on FM, its use on the left-(l)-DLPFC revealed benefits on cognitive performance (Santos et al., 2018), and its use at home was effective in improving pain (Brietzke et al., 2020) and pain catastrophizing (Caumo et al., 2022).

The a-tDCS can modulate cortical and subcortical neural networks, inducing a top-down effect. Its effect on healthy controls (HC) demonstrates that a-tDCS over the l-DLPFC improved digit-span performance (Rottschy et al., 2012; Barbey et al., 2013). However, other studies found that it enhanced digit-span performance only if the stimulus had been paired with an online WM (n-back) task (Hill et al., 2019). Additionally, we showed that the alertness, orienting, and executive control attentional networks are all modulated by a single session of a-tDCS with 2 mA administered to the l-DLPFC in combination with a Go/No-go test (Silva et al., 2017). Besides, studies found that tDCS’s impact on pain and cognitive function is neuroplasticity state-dependent, as indexed by the brain-derived-neurotrophic factor (BDNF) (Santos et al., 2018; Brietzke et al., 2019; da Graca-Tarragó et al., 2019). In the same perspective, earlier studies found that serum BDNF is associated positively with the descending pain modulatory system malfunction (DPMS) (Caumo et al., 2016; Soldatelli et al., 2021) and that the BDNF likely mediates the a-tDCS effect in the improvement of DPMS (da Graca-Tarragó et al., 2019; Beltran Serrano et al., 2020). Hence, substantial evidence supports the critical role of BDNF in synaptic plasticity, learning, and memory (Kowianski et al., 2018), and a decrease of this neurotrophic factor in the hippocampus is related to the worst cognitive performance on memory tasks (Etnier et al., 2015). In this setting, it is reasonable to consider the BDNF as a neural plasticity marker involved in the tDCS effects, either on pain processing or cognitive functions (Cocco et al., 2018; Santos et al., 2018). Within this frame, more in-depth analyses of tDCS action are important to comprehending the molecular and neurophysiological mechanisms subtending tDCS effects on cognitive processes and DPMS dysfunction (Soldatelli et al., 2021). So, comprehension of its impact on the neuroplasticity processes, with the perspective to link them with clinical effectiveness, might help with better use of this technique.

Thus, we determine whether 20 sessions of a-tDCS on the left (l)-DLPFC and cathodal on the right (r)-DLPFC over 4 weeks self-applied at home would be superior to a sham-(s)-tDCS in improving the executive attention, divided attention, working memory, and cognitive flexibility assessed by the Trail Making Test (TMT-B-A) (primary outcome). Additionally, we evaluated its impacts on executive functioning (Controlled Oral Word Association Test; COWAT); the WM (Digits subtest from Wechsler Adult Intelligence Scale; WAIS-III); and quality of life (secondary outcomes). We investigated if the tDCS effects were related to the severity of the DPMS dysfunction at the start of the treatment and with neuroplasticity changes evaluated by the percent change in the BDNF from pre- to treatment end. We hypothesized that a-tDCS could improve cognitive performance more effectively than s-tDCS. Besides, we investigated whether these effects were correlated with the degree of pain processing pathway malfunction as measured by baseline DPMS deficit. We also investigated if the tDCS effects are mediated by changes in the neuroplasticity state, as indexed by serum BDNF.

## Materials and methods

### Study design and eligibility

The trial’s protocol was approved by the research ethics committee at the Hospital de Clinicas de Porto Alegre (HCPA), Brazil. Institutional Review Board IRB (36995020.3.0000.5327 CAAE registry) and Research Ethical Committee registration number 2017-0330. Each patient gave verbal and written consent to participate in this randomized, double-blind, sham-controlled trial. No compensation was given to participants in exchange for their participation.

### Inclusion and exclusion criteria

We included adult females ages 30–65 right-handed if they met the diagnostic criteria of fibromyalgia, according to the American College of Rheumatology (ACR, 2016). They were recruited through newspaper advertisements and recruitment from the outpatient pain clinic at HCPA. The FM diagnosis was confirmed by a Brazilian board-certified pain specialist. To be included, they need to be literate and report a score of at least six on the Numerical Pain Scale (NPS 0–10) on most days of the previous 3 months. Additionally, they should have consented to continue taking their medication during the study at the same doses used during the previous month starting the study. The exclusion criteria comprise the history of brain surgery, a tumor, a stroke, or the implantation of intracranial metal. Additionally, individuals were excluded if they had used illicit drugs during the previous 6 months or had an uncompensated clinical illness (i.e., ischemic heart disease, renal disease, hepatic disease, diabetes mellitus, hypertension, etc.). Rheumatoid arthritis, lupus, autoimmune disease, neurologic, oncologic disease, or COVID symptoms were additional exclusion criteria.

### Sample size justification

Sample size estimation was based on a previous study that tested ten sessions of a-tDCS over the l-DLPFC in non-demented, ambulatory older adult patients on the Trail Making Test (TMT- B-A) (Manor et al., 2018). Our estimation was established using a 2-tailed test for a ratio of 2:1 (a-tDCS vs. s-tDCS on the DLPFC), a type I error of 5%, and a power of 80%. The standard deviation (SD) from the s-tDCS group was used as a reference to estimate the effect size (Manor et al., 2018). For an ES of large magnitude [(*f*) equal to 1.02, considering a pooled standard deviation (SD) at treatment end equal to 34)], the estimated sample size was 30 patients. We included an additional 20% of subjects to account for possible dropouts. Thus, the final sample size was 36 patients (24 in the a-tDCS vs. 12 in the s-tDCS group).

### Randomization

Thirty-six patients were randomized at an allocation of 2:1 to groups a-tDCS or s-tDCS, using random numbers created with the proper software. We employed randomization in three blocks of 12 patients to prevent the possible allocation prediction of the treatment group. Two investigators who were not involved in the patient assessments conducted the randomization before the recruitment stage. The envelopes containing the randomization number were prepared and according to the exterior numerical order, these envelopes were sealed and numbered in order. Research partners not involved in the trial, neither in contact with subjects nor evaluations, opened the envelopes and programmed the devices.

### Blinding

Participants were uninformed of their therapy throughout the entire program (active or sham). Additionally, the allocation was unknown to the research team, the investigators who assisted with patient care, and the people who used the scales. The s-tDCS group’s device was set up to provide 30 s of stimulation throughout the course of 20 min in the beginning, after 10 min, and after the stimulation. Each of these times, the device was set up to automatically switch on and off. By employing this strategy, we concealed the intervention for all research members until the treatment ended.

### Intervention

The anode was placed on the l-DLPFC (F3) and the cathode at the r-DLPFC (F4) by the 10–20 system for EEG). The treatment was administered for five consecutive days over 4 weeks, totaling 20 sessions.

Participants received the programmed device to use at home. For the active a-tDCS, the current applied was 2 mA for 20 min (Carvalho et al., 2018; Brietzke et al., 2020). For sham s-tDCS conditions, the montage was the same as active tDCS. A 30-s ramp-up in intensity from zero to 2 mA was used for a-tDCS and s-tDCS stimulation, as well as a ramp-down for about the same duration, as explained in the blinding session. Using two silicone cannulas attached to 35 cm^2^ (5 × 7 cm) electrodes coated in sponges wet with saline solution, the current was supplied. The gadget was programmed by a single biomedical engineer to provide a set number of stimulation sessions, with a minimum gap of 16 h between each successive session. Details about the protocol can be seen in complementary material and a paper by Santos et al. (2021).

***Treatment protocol with tDCS at home was established according to the standardized protocol described below:*** (1) Visit the facility, (2) Cap size and electrode placement, (3) Training, (4) Compliance with the protocol, proper application, and adverse effects.

***(i) Volunteer who visited the lab as part of the methodology***

**First Visit:** Upon arriving at the laboratory, they provided their written, formal consent, confirmed the diagnosis, completed the sociodemographic questionnaire, underwent the cognitive test, and completed other baseline assessment procedures. They were also provided with information regarding the protocol they will follow.
**Second visit:** The first 20 min of the treatment session were administered, which also included a training session on how to use the device at home.
**Third Visit:** The patient returned the device to the lab after completing the assessment at the end of the treatment, which took place after 4 weeks of tDCS at home.

***(ii) Size of the cap and electrodes’ position, training session, protocol compliance, and adherence***

**FIGURE 1:**
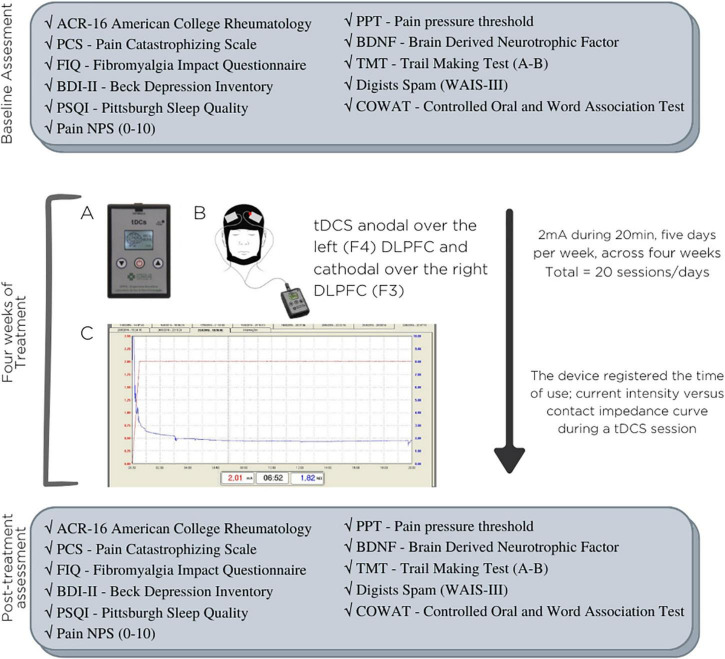
Timeline of the study. **(A)** Home tDCS device; **(B)** position of electrodes on the DLPFC (dorsolateral prefrontal cortex); **(C)** typical curves of current intensity vs. contact impedance during a tDCS session.

(a) *Procedures to choose the size of the cap and electrodes’ position: Following the measurement of* the head circumference, the researcher selected the size of the cap from small (38 cm × 55 cm), medium (39 cm × 57.5 cm), and large (40 cm × 59 cm). The researcher then localized the electrode positions using the 10–20 system for EEG and placed electrodes in the F3 and F4 positions to deliver current to the scalp. The user cannot move the electrodes once they are inside the sponges, so an exact location of the electrode to provide the electric current during stimulation is assured.
(b) *Training session and instructions on how to self-apply the tDCS:* After guiding the participants with the information from the tDCS use at the home manual and answering any questions, we conducted a face-to-face training session in the clinical research facility at HCPA in Porto Alegre, Brazil.
- Patients might access the step-by-step procedure for self-administration of tDCS at the following link (YouTube: https://youtu.be/3Wtji4esOGE).
(c) *Protocol compliance, appropriate use, and record of adverse effects during the sessions of tDCS at home:*
- Participants received instructions to pick a peaceful during the day to administer the therapy session.
- One research team member remotely supervised the first session at home (the second overall). If the participant had questions or issues about the device, they could contact the research team via WhatsApp anytime.
- The researcher in charge of getting in touch with patients did so once a week.
- The tDCS device software recorded every session.
- Additionally, participants were oriented to note any adverse effects in their diary immediately after the session.
(d) *Control of adherence:* An engineer who was not involved in the patients’ treatment oversaw downloading the data stored in the software during the treatment to maintain the study team’s blinding. Such data include records of hour use, time of use, impedance, resistance, and the number of sessions. The timeline of the study is presented in Figure 1.

### Instruments and assessments

Pain scales, psychological assessments, and psychophysical measurements were all performed by two evaluators unaware of the group assignment. The cognitive assessments were conducted by two trained psychologists. The tests were administered with auditory or paper stimuli and oral responses. All evaluators received a specific training, which followed a sequence of steps: ***(i)*** read and study manuals of each test; ***(ii)*** observe the administration of tests by an experienced examiner; ***(iii)*** practice it on volunteers in role-playing sessions, and (***iv***) discuss problems and questions with local experts if was needed. All assessments were performed in a quiet and private area without interruptions after patients received correct and clear instructions for the test in a slow speaking voice.

#### Outcomes

The primary outcome was executive function, defined as TMT Part B minus Part A. The secondary outcomes were working memory, assessed by Digits Span [*Digits subtest from Wechsler Adult Intelligence Scale* (WAIS-III)], verbal fluency (semantic and orthographic), assessed by Controlled Oral and Word Association Test (COWAT), and everyday dysfunction due to FM, assessed by Fibromyalgia Impact Questionnaire (FIQ).

##### Outcome assessments

a. ***TMT—Trail Making Test (TMT A-B)**:* TMT A-B measures working memory, executive attention, cognitive flexibility, split attention, and processing speed (Reitan and Wolfson, 1993; Lezak et al., 2004). The amount of time needed to finish the job and the number of mistakes affect how well you score. Lower performance is indicated by higher scores.
b. ***Digits subtest from Wechsler Adult Intelligence Scale (WAIS-III)**:* The Digit’s subtest consists of eight series of digits presented aloud to the subject and asked to repeat in the same order (forward), and seven sequences that should be repeated in inverse order (backward), each series with a gradual increase in the number of digits (Nascimento, 2004; Wechsler, 2004). The Digits test assesses working memory. Higher scores indicate better performance.
c. ***Controlled Oral Word Association Test (COWAT)***: It is the verbal fluency exam that evaluates both linguistic and executive skills, including cognitive flexibility, strategy use, interference suppression, and reaction inhibition (Hedden and Yoon, 2006; Schinka et al., 2010). Better performance is indicated by higher scores.
d. ***Fibromyalgia Impact Questionnaire (FIQ)*** was proposed by Burckhardt et al., 1991 to assess the quality of life in FM patients. We used the version adapted for use in Brazil (Paiva et al., 2013). The FIQ consists of 10 domains. The items evaluate the patient’s capacity for doing everyday activities as well as their level of weariness, stiffness in the morning, mood, anxiety, and sadness. The scoring cap is 100. Higher scores indicate worse quality of life due to FM symptoms.

##### Psychophysical measurements, depressive symptoms, sleep quality, and serum brain-derived neurotrophic factor

e. The following sequence of procedures evaluated the conditioned pain modulation test (CPM-test): ***First***, we employed the thermo-test placed in the non-dominant forearm on the ventral forearm to define the temperature to produce a score of 6/10 (NPS, 0–10) by an average of three successive measures (T0). ***Second***, patients submerge their dominant hand for 1 min in water at a temperature of 0–1°C. Thirty seconds after they dipped their non-dominant hand in cold water, the non-dominant forearm underwent the QST’s thermo-test. The pain intensity in the thermode region was measured using a scale of 0–10 (QST + CPM-test) (T1). ***Third,*** we calculate the CPM–test score by the difference between the change in NPS 0–10 at the temperature set at 6/10 in the region of thermos-test minus 6 (reference value) (Botelho et al., 2016; Soldatelli et al., 2021). For the analysis, we used CPM-test score as a continuous variable. So higher values indicate lower efficiency of DPMS.
f. ***Pain pressure threshold (PPT)***: To conduct the test, we employed an electronic algometer made by J-Tech Medical Industries in Midvale, Utah, USA. Patients were advised to distinguish between pressure and pain before the assessment began. Patients were told to verbally communicate their pain when it started. At 3–5-min intervals, we took three measurements in succession (da Graca-Tarragó et al., 2019).
g. ***Beck Depression Inventory-II (BDI–II):*** It is a self-applied used to evaluate the severity of depressive symptoms (Gomes-Oliveira et al., 2012).
h. ***The Pain Catastrophizing Scale (B-PCS):*** It is a self-administered questionnaire with 13 items to measure pain-related catastrophizing (Sehn et al., 2012).
i. ***Pittsburgh Sleep Quality Index (PSQI)*** evaluates sleep quality over the previous month. The score with the highest rating represents the poorest sleep.
j. ***Dosage of BDNF serum levels:*** We used the blood collection tubes with gel and clot activator. After centrifuging blood samples, the serum was divided into 0.5 ml aliquots for additional examination. According to the manufacturer’s instructions, sandwich ELISA was used to measure the serum levels of BDNF using monoclonal antibodies specific for the neurotrophin (R&D Systems, Minneapolis, United States). To evaluate the inter-assay variation, two plates per kit were utilized over two distinct days of the same week. The manufacturer’s instructions are followed by protocols. To ascertain serum BDNF, the Enzyme-linked Immunosorbent was employed. The kit’s BDNF lower detection limit is 7.8 pg/ml. Use the ChemiKine BDNF Sandwich ELISA kit, CYT306 (Chemicon/Millipore, Billerica, MA, USA), for the assay (ELISA). GloMax^®^-Multi Microplate Reader from Promega or the Bio-Plex^®^-200 device from Bio-Rad was used to assess optical density for multiplexing assay readings. Using the Bradford method, bovine serum albumin as a standard, we measured the total protein using the standard. The information was presented as pg/mg of protein.

##### Clinical measurements: CSS symptoms, pains score, and analgesic use

k. A standardized questionnaire was used to evaluate demographic information and medical comorbidities. They self-reported diagnoses, medication use, medical procedures, and pain-related problems.
l. The Numerical Pain Scale was used to measure the level of pain (NPS). The NPS scores range from zero (no pain) to maximum agony (10). Patients provided the response to the following question: How severe was your worst pain over the past week?
m. The symptoms of central sensitization were evaluated using the Central Sensitization Inventory for Brazilian Population (CSI-BP). Its 25 items (total score of 0–100) examine urological symptoms, headache/jaw symptoms, mental distress, and physical problems. Higher ratings reflect more severe symptoms. Part B of the CSI-BP also evaluates neurological conditions linked to central sensitization and mental diagnoses (Caumo et al., 2017).
n. If an extra analgesic medication (such as acetaminophen, ibuprofen, or tramadol) was required to treat their pain, they could do so. As rescue analgesia, they may take 500 mg of acetaminophen up to four times daily (QID). They could take Dorflex^®^ (Sanofi Aventis, So Paulo, Brazil; 35 mg orphenadrine citrate coupled with 300 mg dipyrone and 50 mg caffeine) up to three times daily (TID) if their discomfort continued. Patients could utilize tramadol at their highest tolerated daily dose if their discomfort continued.

### Statistical analysis

Continuous and categorical variables were compared using Fisher’s exact test, the chi-square test, and the *t*-test for independent samples. We utilized the Shapiro-Wilk normality test to determine if the continuous variables displayed a normal distribution. We utilized the Mann-Whitney U-Wilcoxon test for comparisons between groups and the Wilcoxon test for comparisons within groups. Furthermore, we used a linear regression model to examine the impact of the treatment. The treatment group was factored into the models (a-tDCS or s-tDCS) and the dependent variables were evaluated by percent change in average [((value post-intervention minus value pre-intervention)/value pre-intervention) *100]. The primary outcome was assessed by the Trail Making Test (TMT-B-A). The secondary outcomes were the following: working memory, verbal fluency (semantic and orthographic), phonemic fluency, and the impact of FM symptoms on quality of life. Since it is known that cognitive measures exhibit substantial individual variability on the same test, and they do not have a reference value to define the severity of cognitive impairment, they utilized the percent change in the average from pre-to treatment end. We restrict the intention-to-treat analysis (ITT) to subjects who had received at least 50% of the total protocol sessions, in the case of 10 sessions. We used a single imputation approach for missing data, replacing missing values with the mean for the outcome variables (Dziura et al., 2013). We used the pool of baseline standard deviation (SD) to calculate the ES using the standardized difference mean (SDM) (mean difference a-tDCS vs. s-tDCS). The ES was considered minor if it ranged (from 0.20 to 0.49), moderate if it ranged (from 0.5 to 0.79), and large if it was equal to 0.8 or over (Kazis et al., 1989). Spearman’s correlation analysis was used to test the correlation between the average percent change (pre-intervention to treatment end) of TMT-B-A, QIF, and serum BDNF according to a-tDCS and s-tDCS groups. All analyses used two-tailed tests with a significance threshold of 5% and were adjusted for multiple comparisons using Bonferroni’s test. SPSS was used to examine the data, version 22.0 (SPSS, Chicago, IL).

## Results

### Demographic and clinical characteristics of the subjects

**FIGURE 2:**
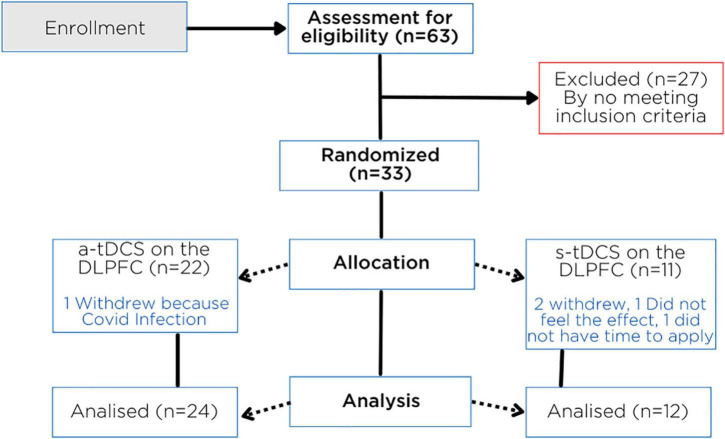
Flowchart showing randomization, allocation, and progress through the study.

We screened 63 patients, and 27 patients didn’t fit the criteria for inclusion. The flow presents the exclusion criteria (Figure 2). This study included 36 patients who were randomly assigned to receive either a-tDCS (*n* = 24) or s-tDCS (*n* = 12). Three patients discontinued therapy—two in the s-tDCS and one in the a-tDCS—one owing to a COVID infection that prevented her from applying the stimulation session, one because she did not feel the effects of the treatment quickly enough, and one because she did not have enough time to apply the treatment. We conducted an ITT analysis including all of them (*n* = 36) since all had completed at least 10 sessions of tDCS.

Table 1 displays the demographic and clinical traits of the patients. There are balanced baseline features between treatment groups.

**TABLE 1:** Epidemiological and clinical characteristics at baseline, according to the treatment group, values are given as the mean (SD) or frequency (*n* = 36).

Characteristics	s-tDCS (*n* = 12)	a-tDCS (*n* = 24)	*P*
Age (years)	46.09 (11.34)	49.18 (8.63)	0.392
Education (years)	12.64 (5.07)	10.23 (3.75)	0.134
American college of rheumatology (ACR) diagnosis criteria score	22.82 (4.36)	23.00 (3.94)	0.901
Smoking (*Yes*)	3	7	0.785
Alcohol (*Yes*)	7	10	0.320
Clinical comorbidity (*Yes*)	50%	70%	0.251
Ischemic cardiac *(Yes)*	0	0	
Hypertension *(Yes)*	0	0	
Diabetes *(Yes)*	1	2	
Hypothyroidism *(Yes)*	2	4	
Asthma *(Yes)*	1	5	
Other *(Yes)*	6	16	
Psychiatric disorder according to the MINI (Yes/No)^†^			
Maniac-depressive disorder *(Yes)*	75%	70%	0.541
Generalized anxiety disorder *(Yes)*	45%	40%	0.552
*Pain, sleep quality and psychological measures*			
Visual analog scale£	8.12 (1.12)	8.65 (1.36)	0.711
Beck depression inventory II (BDI-II)£	24.18 (10.05)	27.91 (11.36)	0.445
Brazilian Portuguese Pain Catastrophizing Scale£	34.36 (10.83)	36.48 (10.94)	0.727
Pittsburgh seep quality index (PSQI)£	11.27 (4.08)	13.45 (4.01)	0.713
Heat pain threshold to produce 6/10 on NPS (oC)£	40.68 (3.31)	40.98 (3.16)	0.793
Change on numerical pain scale during CPM-test^∑^	–1.20 (1.87)	–0.85 (1.89)	0.940
Central sensitization inventory£	58.27 (9.51)	68.59 (13.95)	0.066
Pain pressure threshold (kg/cm^2^/s) ^∑^	1.36 (0.74)	1.49 (1.16)	0.490
Opioid medication user (Yes)^‡^			
Acetaminophen *(Yes)*	1	7	0.151
Dipyrone *(Yes)*	3	5	0.774
Dorflex *(Yes)*	6	9	0.458
Opioid medication user (Yes)^‡^			
Codeine	2	7	0.407
Methadone	1	0	0.333
Tramadol	3	4	0.661
Active central nervous system medication^‡^			
Antidepressant tricyclic or dual *(Yes)*	2	6	0.566
Antidepressant dual *(Yes)*	6	10	0.622
Antidepressants selective serotonin reuptake inhibitors *(Yes)*	2	4	0.853
Pregabalin (Yes)	1	5	0.338
Change on numerical pain scale during CPM-test pre-treatment ^∑^	–1.20 (1.87)	–0.85 (1.89)	0.691
Brain-derived neurotrophic factor (BDNF) (ng/ml) before treatment ^∑^	33.85 (22.17)	49.53 (36.33)	0.192
Brain-derived neurotrophic factor (BDNF) (ng/ml) at treatment end ^∑^	58.53 (48.61)	31.39 (18.32)	0.140
Percent change serum BDNF from pre-intervention to treatment end ^∑^	52.63 (67.82)	–14.48 (60.15)	0.031

^‡^Non-opioid analgesics, opioid analgesics; active central nervous system medications and psychiatric disorder patients could have none or more than one of them. ^†^Patients could have one or more than one psychiatric diagnosis or use one or more medication. ^£^Comparison using t-test for independent samples. ^∑^Comparison using Wilcoxon Mann-Whitney.

### Univariate analysis: Interventions effects within groups on primary and secondary outcomes

The within-group treatment effect in the outcomes: primary [sustained and divided attention assessed through the Trail Making Test (TMT-B-A)] and secondary outcomes [working memory, verbal fluency (semantic and orthographic), phonemic fluency, and impact of fibromyalgia symptoms on quality of life] are presented in Table 2. We showed the mean (standard deviation), median (interquartile 25–75) at baseline, and treatment end, as well as the effect size (ES) according to groups (s-tDCS and a-tDCS).

**TABLE 2:** Univariate analysis: Interventions effects within and between groups on primary and secondary outcomes (*n* = 33).

	s-tDCS (*n* = 12)				a-tDCS (*n* = 24)			
	Mean (SD)	Median (IQR_25–75_)	ES	*P*	Mean (SD)	Median (IQR_25–75_)	ES	*P*
**Primary outcome**								
**Trail making test (TMT-A)**								
Baseline	33.37 (7.94)	26 (9.07; 66.75)	0.36	0.20**^¥^**	38.23 (14.69)	38.58 (17.57; 133)	0.74	0.01**^¥^**
Treatment end	30.91 (10.17)	30.44 (15.19; 50.50)			30.91 (10.17)	29.55 (18.52; 69.90)		
Difference mean (%)	–8.81 (22.08)				–12.55 (23.31)		—	0.64^€^
Trail making test (TMT-B)								
Baseline	60.30 (22.76)	61 (30.62; 103.68)	0.31	0.26**^¥^**	87.45 (33.44)	73.18 (52.81; 176)	0.5	0.00**^¥^**
Treatment end	72.49 (27.85)	66.38 (24.60; 139.5)			74.16 (44.11)	58 (41.04; 110)		
Difference mean (%)	11.73 (37.66)				–15.44 (17.40)		0.72	0.03^€^
**Trail making test (TMT-B-A)**								
Baseline	27.24 (17.61)	30.44 (15.19; 50.50)	0.4	0.32**^¥^**	47.72 (30.25)	29.55 (18.52; 69.90)	0.29	0.04**^¥^**
Treatment end	37.00 (24.40)	35.34 (8.23; 56.56)			40.01 (26.15)	32.51 (10; 85)		
Difference mean (%)	57.20 (45.92)				–18.21 (24.90)		1.06	0.01^€^
**Secondary outcomes**								
**Span digits forward**								
Baseline	7.45 (1.74)	7 (4; 8)	0.46	0.00**^¥^**	7.18 (1.32)	6 (4; 10)	0.35	0.15**^¥^**
Treatment end	6.60 (1.85)	6 (4; 10)			8.11 (2.62)	7 (5;14)		
Difference mean (%)	–12.12 (15.02)		0.16		16.99 (31.43)			0.00^€^
**Span digits for backward**								
Baseline	5.23 (1.45)	4 (3; 8)	0.28	0.07**^¥^**	5.64 (1.43)	6 (3; 12)	0.14	0.38**^¥^**
Treatment end	4.80 (1.54)	4 (3; 8)			6.00 (2.65)	6 (3; 12)		
Difference mean (%)	–7.85 (23.83)		—-		11.90 (36.80)			0.20^€^
**COWAT orthographic**								
Baseline	32.73 (7.86)	34 (17; 49)	0.23	0.08**^¥^**	33.36 (7.45)	34 (21; 43)	0.34	0.01**^¥^**
Treatment end	34.60 (8.07)	35 (19; 51)			36.67 (9.67)	36 (24; 52)		
Difference mean (%)	8.78 (16.26)		—	0.45^€^	12.97 (10.01)			
**COWAT semantic**								
Baseline	17.91 (4.28)	16 (12; 27)	0.02	0.95**^¥^**	15.10 (3.68)	15 (10; 26)	0.68	0.01**^¥^**
Treatment end	17.78 (5.83)	17 (11: 28)			17.59 (3.86)	18.50 (10; 24)		
Difference mean (%)	4.39 (29.83)				13.93 (24.29)		0.31	0.04^€^
**Fibromyalgia impact questionnaire (FIQ)**								
Baseline	71.89 (7.55)	72.44 (55.87; 82.02)	0.65	0.02**^¥^**	76.99 (9.80)	76.13 (61.93; 98.73)	1.15	0.00**^¥^**
Treatment end	64.73 (10.94)	67.02 (47.72; 81.93)			58.42 (15.53)	62.19 (26.10; 83.23)		
Difference mean (%)	–10.30 (9.78)				–24.76 (15.71)		3.58	0.01^€^

^¥^Comparisons within group by Wilcoxon test. ^€^comparisons between groups by Mann-Whitney U-Wilcoxon. Difference mean average percent change [((value post-intervention minus value pre-intervention)/value pre-intervention) *100]. Effect size (ES) (Mean difference a-tDCS vs. s-tDCS)/Pooled standard deviation]. The ES was defined as small if lower than 0.20–0.49; moderate if between 0.50 and 0.79; and large if larger than 0.80.

### Primary outcome: Impact of transcranial direct current stimulation in the executive attention, divided attention, and working memory by trail making test-B-A

**FIGURE 3:**
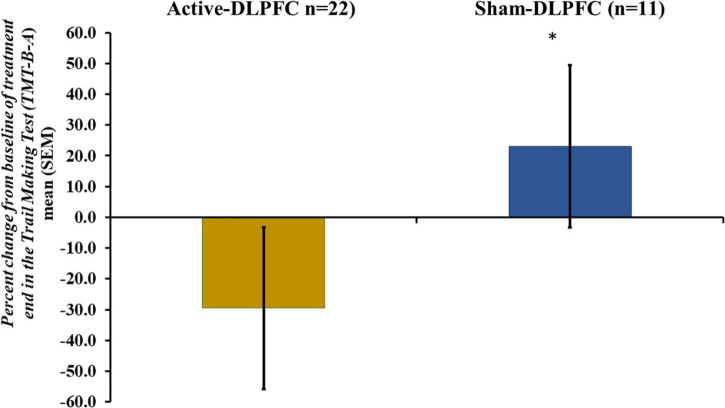
Mean of percent changes of averages from the pre-intervention period to treatment end period of the total score in the Trail Making Test (TMT-B-A). Error bars indicate the standard error of the mean (SEM). Asterisks (*) positioned above symbols indicate significant differences (*p* < 0.05) between groups (a-tDCS and s-tDCS).

The GLM revealed a main effect for treatment assessed through the Trail Making Test (TMT-B-A) (Wald χ^2^ = 6.17; Df = 1, *P* = 0.013). The TMT-B-A score was adjusted by the multivariate analysis presented in Table 3 revealed that the a-tDCS reduced the total score in the Trail Making Test (TMT-B-A) to –29.53 (8.89) compared to an increase in the scores in the s-tDCS 23.09 (16.32) as shown on Figure 3. The ES based on the SDM of a-tDCS vs. s-tDCS was large [–1.48, confidence interval (CI) 95% = –2.07 to –0.90]. It is important to realize that a reduction in the scores of the TMT-B-A indicates better cognitive performance.

**TABLE 3:** Effect of treatment on TMT-B-A (primary outcome).

Cognitive tests	Beta	SEM	95% CI	Wald χ^2^	df	*P*
**Trail making test (TMT-B-A)**						
(Intercept)	25.836	17.992	(–9.42 to 61.10)	2.062	1	0.151
Active-tDCS (*n* = 24)	–48.391	19.472	(–86.55 to –10.22)	6.176	1	0.013*
Sham-tDCS (*n* = 12)	0^reference^					
Scores on NPS (0–10) during CPM-test at baseline	7.190	3.4483	(0.43–13.94)	4.347	1	0.037*
Change on BDNF pre- to post-treatment (%)	0.501	0.1969	(0.11–0.89)	6.483	1	0.011*

Data present the comparisons between groups on the percent changes from pre-intervention to treatment end (*n* = 36). *Represents a difference significant from a *P*-value < 0.05. CPM-test, conditioned pain modulation; NPS, numerical pain scale; BDNF, brain-derivate neurotrophic factor.

According to the analysis presented in Table 3, the effect of a-tDCS on TMT-B-A was positively correlated with the severity of dysfunction in the DPMS at baseline. For the study of the DPMS function, we used the NPS (0–10) as a continuous variable. Thus, higher scores indicate a lower efficiency of the DPMS. Also, the performance in the TMT-B-A was positively associated with more significant decreases in the serum BDNF from pre-intervention to treatment end. In other words, more considerable reductions in the TMT-B-A in the a-tDCS are associated with a more remarkable decrease in the BDNF at the treatment end.

### Secondary outcomes: Impact of transcranial direct current stimulation on working memory (digits subtest from Wechsler Adult Intelligence Scale), cognitive flexibility (controlled oral word association test), and quality of life

The GLM revealed a main effect for treatment of the a-tDCS effects compared to s-tDCS to improve the performance in the working memory, verbal fluency (semantic and orthographic), phonemic fluency, and impact of fibromyalgia symptoms on quality of life. Data are presented in Table 4.

**TABLE 4:** Effect of treatment on sustained attention, working memory, verbal fluency, phonemic fluency, and quality of life.

Cognitive tests	Beta	SEM	95% CI	Wald χ^2^	df	*P*
**Span digits forward**						
(Intercept)	6.956	8.20	–9.12 to 23.04	0.71	1	0.397
Active-tDCS (*n* = 24)	22.19	8.85	4.84–39.54	6.28	1	0.012*
Sham-tDCS (*n* = 12)	0^reference^					
Change on BDNF pre- to post-treatment (%)	0.045	0.04	–0.02 to.11	1.67	1	0.196
Responders vs. non-responders to CPM-test	12.41	7.60	–2.49 to 27.31	2.66	1	0.103
**Span digits backward**						
(Intercept)	10.42	11.27	–11.66 to 32.53	0.85	1	0.355
Active-tDCS (*n* = 24)	16.65	12.51	7.87–41.18	1.77	1	0.183
Sham-tDCS (*n* = 12)	0^reference^					
Change on BDNF pre- to post-treatment (%)	0.024	0.085	–0.14 to 0.19	0.083	1	0.773
Change on NPS (0–10) during CPM-test at baseline	0.423	10.80	–20.75 to 21.60	0.002	1	0.969
**COWAT orthographic**						
(Intercept)	8.24	5.71	–2.98 to 19.44	2.07	1	0.150
Active-tDCS (*n* = 24)	0.31	6.13	–11.72 to 12.34	0.003	1	0.959
Sham-tDCS (*n* = 12)	0^reference^					
Change on BDNF pre- to post-treatment (%)	0.034	0.02	–0.01 to 0.08	1.93	1	0.164
Change on NPS (0–10) during CPM-test at baseline	3.04	5.51	–7.76 to 13.85	0.30	1	0.581
**COWAT semantic**						
(Intercept)	7.52	10.38	–12.82 to 27.87	0.53	1	0.468
Active-tDCS (*n* = 24)	23.44	11.20	1.49–45.40	4.38	1	0.036*
Sham-tDCS (*n* = 12)	0^reference^					
Change on BDNF pre- to post-treatment (%)	–0.05	0.044	–0.13 to 0.04	1.12	1	0.290
Change on NPS (0–10) during CPM-test at baseline	3.49	9.621	–15.36 to 22.35	0.13	1	0.716
**Fibromyalgia impact questionnaire (FIQ)**						
(Intercept)	–13.34	5.51	–24.14 to -2.55	5.86	1	0.015
Active-tDCS (*n* = 24)	–13.96	5.88	–25.50 to -2.42	5.62	1	0.018*
Sham-tDCS (*n* = 12)	0^reference^					
Change on BDNF pre- to post-treatment (%)	–0.014	0.0233	–0.06 to 0.03	0.37	1	0.539
Change on NPS (0–10) during CPM-test at baseline	10.06	5.27	–0.27 to 20.39	3.64	1	0.056

Data present the comparisons between groups on the percent changes from pre-intervention to treatment end (*n* = 36). *Represents a difference significant from a *P*-value < 0.05. CPM-test, conditioned pain modulation; NPS, numerical pain scale; BDNF, brain-derivate neurotrophic factor.

### Secondary outcomes’ analysis: Trail making test, serum brain-derived neurotrophic factor and quality of life after treatment ends

In the a-tDCS, there is a moderate and positive correlation between the TMT-B-A at treatment end with changes in serum BDNF [(Rho = 0.57, confidence interval (CI) 95% = 0.28–0.76; *P* = 0.01]. In contrast, in s-tDCS the correlation between these two variables was not significant [(Rho = 0.25, confidence interval (CI) 95% = –0.1 to 0.55, *P* = 0.70]. In the a-tDCS, the TMT-B-A scores at the treatment end showed a positive and moderate correlation with the scores related to FM symptoms on quality-of-life [(Rho = 0.66, confidence interval (CI) 95% = 0.4–0.82; *P* = 0.001]. In contrast, in s-tDCS the correlation between these two variables was not significant [(Rho = 0.20, confidence interval (CI) 95% = –0.15 to 0.51; *P* = 0.60]. Such non-parametric correlations showed that patients who received a-tDCS showed a remarkable cognitive performance improvement. In the same way, they presented a more considerable reduction in serum BDNF, which was moderate and positively correlated with improved cognitive performance and improvement of symptoms that impact the quality of life.

### Assessment of adverse events and safety

The adverse effects comprising headache, tingling, burning, redness, and itching were not significantly different between a-tDCS and s-tDCS (see Table 5). Both groups experienced comparable mild side effects (a-tDCS or s-tDCS). Many side effects were rated as light, and no patients discontinued therapy because of uncomfortable side effects.

**TABLE 5:** Side effects are presented as percentage (%), and the incidence or severity of side effects classified as absent, mild, moderate, and severe (*n* = 36).

			Severity of symptoms (%)			
	Group	Absent	Mild	Moderate	Severe	*P*-value
Headache	a-tDCS = 24	45%	15%	35%	5%	0.26
	s-tDCS = 12	40%	30%	10%	20%	
Tingling	a-tDCS = 24	45%	25%	20%	10%	0.57
	s-tDCS = 12	50%	20%	30%	0%	
Burning	a-tDCS = 24	35%	25%	25%	15%	0.50
	s-tDCS = 12	60%	10%	20%	0%	
Redness	a-tDCS = 24	80%	10%	10%	0%	0.16
	s-tDCS = 12	100%	10%	0%	0%	
Itching	a-tDCS = 24	45%	20%	25%	10%	0.61
	s-tDCS = 12	50%	20%	30%	0%	

To determine protocol compliance and adherence, we verified the co number of valid sessions by the software’s records. The mean number of sessions administered in the a-tDCS group the mean (SD) was 17.35 (3.57); median 19; IQ_25–75_ (10; 20) and in the s-tDCS the mean (SD) was 17.30 (2.54), median 17.5; IQ_25–75_(14; 20). There would be 440 sessions in the group that received a-tDCD (*n* = 22), but we logged 370 valid sessions (84.09%). There were 220 legitimate sessions in the group that received s-tDCS (*n* = 11), while there would have been 178 sessions (80.09%). We recorded 548 valid sessions out of 660 scheduled sessions in all samples, considering a-tDCS and s-tDCS.

## Discussion

This trial demonstrated that the current protocol of home-based a-tDCS compared to sham for 4 weeks of stimulation over the l-DLPFC improved the cognitive functions assessed by TMT-B-A (executive attention, divided attention, W.M., and cognitive flexibility). Additionally, they provide evidence of the a-tDCS benefits to improving secondary outcomes, including W.M., verbal semantic fluency, phonemic fluency, and the impact of F.M. symptoms on quality of life. The a-tDCS effects on the cognitive tests were positively correlated with the percent changes in averages from pre-treatment to treatment end of the BDNF. Also, the severity of dysfunction of DPMS at baseline predicted more remarkable a-tDCS effects in the cognitive impairment. Besides, we found that the reduction in the BDNF related to the a-tDCS is related to improving symptoms due to F.M. The study had a dropout rate of 10%, mainly due to restrictions on circulation in the streets instituted during the COVID-19 pandemic. Mild to moderate adverse events were more common in the active tDCS group, particularly skin tingling, burning, and itching, and the global adherence was 83.03%.

This trial has key methodological differences compared to previous studies on tDCS to improve the cognitive impairment in F.M. We used a home-based tDCS device that enabled a considerably higher number of sessions. Hence, until we can be known, this is the study that evaluated the highest number of sessions used to improve cognitive performance at home. This is particularly relevant since preliminary evidence points to the increased efficacy of tDCS with more extended periods of treatment (Brunoni et al., 2016; Castillo-Saavedra et al., 2016; Brietzke et al., 2020). Additionally, the need for daily visits to clinics or hospitals has always been a significant challenge for using tDCS in the clinical context (Charvet et al., 2020; Salehinejad et al., 2021). Thus, the home-based device opens a new window of opportunity, especially for subjects with physical or cognitive disabilities that hinder their access to the clinical center. So, these results corroborate other previous studies which found that the a-tDCS on the DLPFC might activate regions associated with pain processing, such as the anterior cingulate cortex (ACC) cortex, the primary somatosensory cortex (S.I., SII), insula, and thalamus (Apkarian et al., 2005; Leknes et al., 2008; Bushnell et al., 2013). The fact that pain, as well as attention, share the same cognitive network results in a hindrance to having an efficient cognitive system. Therefore, pain may impair voluntary attentional systems and associated executive functions (Eccleston and Crombez, 1999; Bell et al., 2018). A-tDCS can alter the electrical activity of specific brain regions, encourage cortical plasticity, and enhance functional connections in the area that is being treated, improving pain modulation and quality of life. So, the a-tDCS’s effect modulates neuronal membrane potential on cortical and subcortical neural networks involved in cognitive functions and pain processing. This effect corroborates the results of an earlier trial in a single session of tDCS with 2 mA, applied to the DLPFC cortex in F.M., which found improvements in the function of neural networks involved in spatial and executive attention, as well as a reduced perception of pain (Silva et al., 2017). Another trial also observed the benefit of eight tDCS sessions paired with cognitive training on working memory, verbal fluency, and immediate and delayed memory (Santos et al., 2018). Besides, the current findings are aligned with previous studies in patients with a depression diagnosis, which found that a-tDCS over the DLPFC reduced depressive symptoms and other symptoms linked to inappropriate emotional functioning (Brunoni et al., 2017). As well it reduced pain catastrophizing (Caumo et al., 2022) and improved cognitive functions (e.g., decision-making) (Dixon et al., 2017). According to the literature, this effect can be related to top-down control that up-regulates reactions to positive emotional stimuli (Grimm et al., 2008; Goldin et al., 2009). The ability of a-tDCS on the l-DLPFC to alleviate cognitive abnormalities, notably hypoactivity in the l-DLPFC and hyperactivity in the r-DLPFC, may be one of the potential mechanisms behind these processes and related to its impact on cognitive impairment. This hypothesis is supported in a study that assessed how inter-hemispheric connectivity conservation could have cognitive implications (Krupnik et al., 2021). So, the current result might contribute to a greater understanding of the tDCS effect on brain function.

According to the CPM-test, the severity of DPMS inhibitory dysfunction predicts a remarkable a-tDCS effect compared to s-tDCS on improving cognitive function. This finding suggests that the a-tDCS impact on the outcomes has been more evident in more severe diseases. These findings demonstrate that there is an interaction between the spine-bulbospinal loop and the neural network of cortical areas from an integrative approach. They support the notion that the DPMS and the brain networks involved in cognitive processing have similar neurobiological workings. According to the research, the DLPFC is, therefore, a crucial brain area for modulating the experience of pain. The benefits of using the l-DLPFC as a target area to modulate pain corroborate meta-analysis data that showed the a-tDCS on pain with a moderate E.S. (0.54) (Zortea et al., 2019). Besides, the DPLCF as a target area for improving cognitive performance finds support in data that links prefrontal cortex function with a decline in cognitive abilities (Wen et al., 2011; Wiseman et al., 2018), as well as with the impact of a-tDCS on the l-DLPFC in W.M (Santos et al., 2018). Thus, it is plausible that the cognitive impairment in chronic pain encompasses dysfunctions in neural networks in brain areas with a central role, either in pain (Staud and Spaeth, 2008; Bosma et al., 2016) or in cognition, such as the insula, ACC, and PFC (Nijs et al., 2021). Other studies showed the benefits of a-tDCS on l-DLPFC are supported by improvement in the W.M. and clinical and experimental pain either by repetitive transcranial magnetic stimulation (r-TMS) (Graff-Guerrero et al., 2005; Borckardt et al., 2007) or a-tDCS (Santos et al., 2018). This information reveals that the downstream regulating circuits, including the anterior insula, hypothalamus, periaqueductal gray substance, nucleus accumbens, and rostroventral medulla, are involved in the processes encompassing the effects of a-tDCS on the l-DLPFC (Wager et al., 2013).

The effect of repetitive sessions of a-tDCS has been attributed to the induction of use-dependent neuroplasticity, which is related to “synaptic learning” and long-term changes, which resemble glutamatergic synapses’ long-term potentiation (LTP) or long-term depression (LTD) (Nitsche and Paulus, 2000, 2001). The activity level of underlying neuronal populations at stimulation time is a potentially important mediator of the effect of tDCS on brain function. This is further corroborated by the fact that the impact of tDCS to improve cognitive performance is positively correlated with the neuroplasticity state, according to the percent change in serum BDNF from pre- to treatment end. This discovery aids in understanding how a-tDCS affects faulty neuroplasticity since it can alter mechanisms that include strengthening glutamatergic synapses while weakening GABAergic synapses (Coull et al., 2005). The relationship of serum BDNF to predict the a-tDCS was found in our previous studies with F.M. with a-tDCS applied to the DLPFC in work memory (Santos et al., 2018). In a study with a similar montage, the baseline BDNF predicted the tDCS effect on daily pain scores after sixty sessions of tDCS self-applied at home (Brietzke et al., 2020). Besides, in the postoperative recovery of the hallux valgus surgery, the liquor BDNF after two a-tDCS sessions was associated with lower pain scores and disability due to pain 7 days after surgery (Ribeiro et al., 2017). According to earlier studies, it has been found that there is higher serum BDNF in FM compared to other chronic pain and healthy subjects (Deitos et al., 2015; Stefani et al., 2019). Therefore, the decrease in serum BDNF in the a-tDCS group compared to the sham group and the improvement in cognitive function suggest that the intervention counter-regulated the FM-related dysfunctional neuroplasticity. Despite the relevance of this finding to indicate how much the effect of this therapy might help to improve maladaptive neuroplasticity, this result should interpret sparingly because it is an indirect measure of the neuroplasticity phenomenon.

Our findings should be viewed considering some limitations. *First*, although patients received comprehensive training in using the device, no remote monitoring of sessions was performed. Therefore, there should be caution in direct comparison with studies with supervised electrode placement and exposure supervision. *Second*, we included only females to remove the potential bias due to sex since it has been found that in women, the a-tDCS over DLPFC produces a higher current flow to the frontal regions (Russell et al., 2017) and better performance in cognitive tasks than in men (Martin et al., 2017). *Third*, our findings are consistent with past research that supported this method of self-application for prolonged tDCS use at home. We also see similar outcomes to studies in which the therapy was administered under close observation (Santos et al., 2018). *Fourth*, the tDCS system used in the current study provides an effective technical solution that enables medical engineers who were not involved in the patients’ care to program the tDCS system by the randomization sequence to ensure that all members of the research team and patients are blinded. *Fifth*, in this study, high adherence was observed by the records of devices in use, like those obtained in real-life environments. *Sixth*, despite the randomization processes permit to have balanced groups of a-tDCS and s-tDCS on cognitive performance may be confounded by other variables, such as psychiatric comorbidities (i.e., depression, anxiety, and sleep disturbance) (Austin et al., 2001; Airaksinen et al., 2005; Castaneda et al., 2008), or medication use, particularly opioids, which may lead to cognitive side effects that cannot be controlled entirely (Ersek et al., 2004). *Seventh*, there is no standard battery of neuropsychological tests for cognitive function assessment in chronic pain. So, the literature has recommended that the cognitive assessment in FM. should include tests to evaluate attention and W.M., complex psychomotor speed, and executive functioning (Kravitz and Katz, 2015). *Eight*, the allocation sequence was developed following a standard format described in the scientific literature. Table 1 reveals that most baseline variables are balanced across groups, indicating that randomization equilibrated the groups (a-tDCS and s-tTDC). Although the CSS baseline disparity between a-tDCS and s-tDCS may be attributable to random chance, it is not possible to rule out the effect of regression on the mean. That is, a higher score on the outcome initial might tend to be lower upon subsequent measurement. *Nine*, we decide by allocation 2:1 based on the rationale that fibromyalgia has important suffering. Based on the argument that fibromyalgia causes significant long-term discomfort, we allocate 2:1 since, if a lower number of participants in the sham group, we may treat more individuals actively, leading to increased adherence. Additionally, the higher sample size in the active group increases the ability to identify side effects (Dumville et al., 2006; Hey and Kimmelman, 2014). Finally, with an adherence rate of more than 85% to sessions in both a-tDCS and s-sham tDCS, we adopted a strict and reproducible technique to demonstrate the efficacy and viability of t-DCS at home. However, further studies must explore if neurophysiological measures, such as EEG records, might help to shed light on the specific modulated cognitive processes by the intervention. Another aspect is to allow a more focal target area of the stimulation using multichannel tDCS montages.

These findings revealed that daily treatment with a home-based tDCS device over l-DLPFC compared to sham stimulation over 4 weeks improved the cognitive impairment in F.M. The a-tDCS at home was well-tolerated, underlining its potential as an alternative treatment for cognitive dysfunction. Besides, the a-tDCS effect is related to the severity of DPMS dysfunction and changes in neuroplasticity state.

## Data availability statement

The raw data supporting the conclusions of this article will be made available by the authors, without undue reservation.

## Ethics statement

The Research Ethics Committee approved the protocol for this trial at the Hospital de Clínicas de Porto Alegre (HCPA), Brazil. The patients/participants provided their written informed consent to participate in this study.

## Author contributions

WC, RA, PVS, and FF had substantial contributions to the conception and design of the work. CA, LR, PRS, DS, IL, FF, and WC drafted the work and revised it critically for important intellectual content. WC and FF agreed to be accountable for all aspects of the work in ensuring that questions related to the accuracy or integrity of any part of the work that were appropriately investigated and resolved. All authors agreed and approved the final version of this work.
